# Plasma sCD36 as non-circadian marker of chronic circadian disturbance in shift workers

**DOI:** 10.1371/journal.pone.0223522

**Published:** 2019-10-24

**Authors:** Daniella van de Langenberg, Jelle J. Vlaanderen, Martijn E. T. Dolle, Aase Handberg, Roel C. H. Vermeulen, Linda W. M. van Kerkhof

**Affiliations:** 1 IRAS, Institute for Risk Assessment, Utrecht University, Utrecht, the Netherlands; 2 RIVM, National Institute for Public Health and the Environment, Bilthoven, the Netherlands; 3 Department of Clinical Biochemistry, Aalborg University Hospital, Aalborg, Denmark; 4 Department of Clinical Medicine, Faculty of Medicine, Aalborg University, Aalborg, Denmark; Karlsruhe Institute of Technology, GERMANY

## Abstract

Shift work induces chronic circadian disturbance, which might result in increased health risks, including cardio-metabolic diseases. Previously, we identified sCD36 as a potential non-circadian biomarker of chronic circadian disturbance in mice. The aim of the current study (n = 232 individuals) was to identify whether sCD36 measured in plasma can be used as a non-circadian marker of chronic circadian disturbance in humans, which would allow its use to measure the effects of interventions and monitoring in large-scale studies. We compared levels of plasma sCD36 of day workers with recent (< 2 years) and experienced (> 5 years) night-shift workers within the Klokwerk study. We detected no differences in sCD36 levels between day workers and recent or experienced night-shift workers, measured during a day or afternoon shift. In addition, sCD36 levels measured directly after a night shift were not different from sCD36 levels measured during day or afternoon shifts, indicating no acute effect of night shifts on sCD36 levels in our study. In summary, our study does not show a relation between night-shift work experience (recent or long-term) and plasma levels of sCD36. Since we do not know if and for which time span night-shift work is associated with changes in sCD36 levels, and our study was relatively small and cross-sectional, further evidence for an association between chronic circadian disruption and this candidate biomarker sCD36 should be gathered from large cohort studies.

## Introduction

Night-shift work is an inevitable part of our 24/7 society. Surveys in Europe estimated that approximately 19% of the workers in the European Union (EU) work regularly at night and 17% are involved in night-shift work with permanent or rotating shifts [1]. Night-shift work interferes with timings of normal daily activities [2], disrupting circadian rhythms of many physiological processes. Chronic disturbed circadian rhythms result in increased risks for long-term health effects, such as obesity, cardio-metabolic diseases [3] and potentially breast cancer [4,5].

Current ‘classic’ circadian biomarkers, such as melatonin, cortisol and body temperature, are used to acquire detailed insight into acute disturbances in circadian rhythms in humans [6], based on multiple measurements around the clock or time-standardized measurements. In addition to these ‘classic’ circadian markers, approximately up to 10% of the transcribed genes is under circadian control, providing additional rhythmic markers to estimate a person’s ‘body time’ that could overcome certain limitations [7]. Levels of ‘classic’ circadian markers levels depend strongly on when in the circadian cycle the measurements were taken [8,9]. Because of their short half-life, these ‘classic’ circadian markers provide only limited insights into the cumulative biological effects of chronic circadian disturbance, which occurs during shift work [7–9]. A non-circadian biomarker would enable monitoring circadian disturbances in large-scale human cohort studies and could for example be used to explore the effectiveness of preventive measures in minimizing health risks in intervention studies or research long-term effects. In addition, a promise of a suitable biomarker for circadian disruption would be the ability to pick up biological differences in response to a similar history of night-shift work experience.

Previously, we have investigated universal non-rhythmic biomarkers for chronic circadian disturbance in mice liver [10] using a transcriptomic approach. In this previous mice study, we identified one candidate biomarker that is also present in human blood: sCD36 [10]. Mice serum samples showed statistically significant increased levels of sCD36 14 days after chronic circadian disruption ended, suggesting a relatively long-term effect [10].

CD36 is a class B scavenger receptor present in many cell types and tissues. CD36 is also present in plasma, termed sCD36 (soluble), and its levels parallel those in multiple tissues [11]. CD36 has several functions related to fatty acid regulation and immune responses (dependent on cell type and ligand specific-binding), such as transmembrane transportation of LDL, being an adhesion molecule, being a platelet activator, binding and uptake of long chain fatty acids. Previously, sCD36 has been identified as a biomarker for pathological conditions associated with metabolic dysregulation, including type 2 diabetes mellitus [12], metabolic syndrome [13], obesity [14] and cardiovascular diseases such as atherosclerosis [15,16]. Interestingly, *Clock*^*Δ19/Δ19*^ mutant *Apoe*^*-/-*^ knock out mice show an increased expression of sCD36 in macrophages compared to wild type clock *Apoe* knock out mice, indicating that circadian regulation is an important factor in sCD36 expression and its link to atherosclerosis [17].

The aim of this study was to investigate if the candidate marker sCD36 is a marker of both acute and chronic circadian disturbance due to night-shift work in humans. CD36 is identified as a potential non-rhythmic biomarker for chronic circadian disruption in mice [10], following-up this finding, we compared levels of plasma sCD36 of day workers, short-term and long-term night-shift workers within the Klokwerk study, an observational study among female nurses. We split up short-term and long-term night-shift workers to assess whether we could detect difference in response between these two groups. Underlying processes for such differences might include a potential adaptation or a selection process of individuals better capable handling night shift work among more experienced night-shift workers, or it might reflect a cumulative effect of long-term shift work.

## Material and methods

### Study design and sample collection

Study design, recruitment and inclusion criteria are described elsewhere [18]. In brief, we included female nurses and paramedic staff aged 18–67 years in our study. We included participants that had recently (less than 2 years ago) started working in night shifts, had long-term experience of working in night shifts (over 5 years), or had not worked in nigh-shifts in the last five years before recruitment (defined as ‘day-workers’). We defined night-shift work as working in a rotating-shift schedule including night shifts at least once every six weeks. A night shift was defined as working at least one hour between midnight and 6:00 hour. ‘Day work’ was defined as all work that does not cover the definition of a ‘night shift’, and besides traditional working hours during the day, this also includes morning and afternoon shifts. To participate in the study, subjects had to agree to blood sampling and filling out the questionnaires. We excluded current and former smokers (who quit smoking >6 months before study inclusion and/or smoked <100 cigarettes during lifetime were considered nonsmokers [19]). We excluded participants that were pregnant (or had been in the six months before inclusion); were undergoing fertility treatment; had ever been diagnosed with cancer (excluding non-melanoma skin cancer), had high blood pressure (using beta-blockers), or diagnosed cardiovascular disease; used melatonin supplementation or medication for chronic disorders including diabetes. We approached potentially eligible participants in five Dutch hospitals. All participants signed an informed consent. Inclusion of the participants took place between February 2015 and February 2017. The study was approved by the Institutional Review Board of the University Medical Centre Utrecht, the Netherlands (14-611D, NL51501.041.14). All participants signed an informed consent.

We collected non-fasted blood samples during time-periods in which study participants did not conduct night-shift work (defined as a day session). During these day sessions, samples were ideally collected on days and times when participants were least disrupted by their working schedule: while working afternoon shifts (*i*.*e*. their working schedule did not affect their preferred time of waking up) and as long as possible after the last period working night shifts. We aimed to collect blood samples twice per participant during day sessions (during two separate sessions). In addition, for night-shift workers we collected one blood sample in the morning immediately after a night-shift session. Blood sampling occurred using the standard phlebotomy technique of venipuncture of forearms veins. EDTA plasma samples were stored at -80°C until further processing.

### Covariates

We acquired participant characteristics including age, education, marital status, night-shift work experience (including history of night-shift work, duration in total years of shift work and intensity in number of shifts per month), and recent infections by a questionnaire. Chronotype was self-assessed by the participants using an item of the Horne-Ostberg scale [20] on diurnal preference with five categories (obvious morning preference, more morning than evening preference, more evening than morning preference, obvious evening preference, no specific type). This self-assessment of one’s chronotype gave a similar result (R-square of 0.8) to the 19-item questionnaire in a validation study by Roenneberg *et al*. [21]. We measured weight and height and calculated body mass index (BMI) dividing weight in kilograms by height in meters squared. We recorded the time and date of blood sampling for each collected blood sample. In addition, we recorded time of waking up for a subset (n = 204 samples) of the study population (n = 355 samples). This allowed us to express time of blood sampling as ‘wall-clock time’ and as ‘time since waking up’. (Absence of) circadian variation in plasma sCD36 levels was assessed graphically and by assuming a 24hr cyclical trend and assessing the linear effect of ‘time of blood sampling’ on the cyclical variation by calculating “sin(2*pi* time of blood sampling /24)” and “cos(2*pi* time of blood sampling /24)”, and incorporating both functions in the regression model. Meal timing was assessed using 24hr logs for a subset (n = 205 samples). We asked our study participants to specify their timing of eating and type of nutrition (product and quantity). We calculated the timing of last meal before blood draw.

### sCD36 analysis

We measured plasma CD36 concentrations by an in-house ELISA assay as previously described [22]. In brief, a pool of EDTA plasma was used to produce a standard concentration curve, and phosphate-buffered saline served as background. Absorptions were calculated relative to the standard EDTA plasma pool and expressed as relative units. Internal controls consisting of another EDTA plasma pool were run in two dilutions in duplicates on each plate. Following a standardized freeze-thaw procedure [23], samples were run in two dilutions, and results are given as mean concentrations. Analysis was blinded; however, multiple samples from an individual were always analyzed in one run. Analytical runs were accepted if the internal controls were within ± 1.5 SD from mean, however, the majority were within ± 1 SD. Mean intra-assay coefficient of variation of duplicates was 7%, and total day-to-day assay coefficient of variation was 17.5% (internal controls).

### Data management

Less than 10% of the covariate-values were missing, which we imputed using “multivariate imputation by chained equations” (MICE) package in R (‘pmm-method’) with a single iteration [24]. We included information on the missing data pattern (S1 Appendix). Two sCD36 measurements were identified as outliers and the corresponding observations were removed from the database. One of these observations was identified as an outlier due to long fasting period prior to drawing blood (15 hours).

### Statistical analysis

We used a box plot to show sCD36 concentrations in samples collected during a day shift in day workers, recent night-shift workers (<2 years of night-shift-work experience), and experienced night-shift workers (≥5 years employed in a night-shift-work job), and used a non-parametric Kruskal-Wallis test for significant differences in the means between these groups. Similarly, we used a box plot to show log-transformed sCD36 concentrations of samples collected during a night-shift session and samples collected during a day-shift session among night-shift workers and used a non-parametric Wilcoxon test for significant differences between these groups.

We used a scatter-plot to illustrate (wall-clock) the potential dependency of sCD36 levels on timing of blood sampling for night-shift workers during a night-shift session, night-shift workers during a day-shift session, and day-workers during a day-shift session. In addition, we plotted sCD36 levels in blood versus the difference of timing in minutes between the last meal >50 kcal and timing of blood draw (S2 Appendix).

We conducted linear-regression mixed models to assess differences between day workers and night-shift workers in log-transformed sCD36 concentrations in samples collected during a day shift. Subject ID was included as random variable. In model 1, analyses were corrected for age, BMI, recent infection, season (winter vs. summer time), and chronotype. We assessed the influence of the corrections for BMI, since circulating sCD36 is associated with obesity [25]. In addition, since the timing of blood draw was not standardized, we further adjusted analyses for blood sampling time (wall-clock time), and time since waking up as a proxy for an individual’s relative biological time. Finally, we adjusted our analyses for chronotype, since night workers describe themselves more often as evening types than day workers. In addition, we conducted analyses among morning types only, among evening types only, among recent night workers and controls, among experienced night workers and controls, among overweight individuals (BMI ≥25), and among non-overweight (BMI <25). We provided additional estimates from multiple regression of associations between these covariates and sCD36 concentrations in S3 Appendix. To be able to exclude a possible effect of night-shift work in the past, we also analyzed the effect of night-shift work in a subset where control participants whom previously performed night work were excluded (S4 Appendix. Analyses using a subset of controls whom did not previously worked night shifts). To further research the variability due to night-shift work experience, we included additional analyses regarding duration in total years of shift work and intensity in number of shifts per month and CD36 concentration (S5 Appendix).To estimate the proportion of the total variance in sCD36 levels that was due to between-worker variability we calculated the Intraclass Correlation Coefficients (ICC_worker_).

To investigate if sCD36 levels are related to acute effects of working in a night shift within workers we performed a second set of analyses, model 2. In this analysis, we assessed the difference in log-transformed sCD36 concentrations between samples collected during a night-shift session and samples collected during a day shift session among night-shift workers, using the same models and sensitivity analyses as described above.

## Results

### Description of the study population

Table 1 provides an overview of the study population that consisted of 140 night-shift workers and 89 day workers, contributing 355 blood samples in total. For a proportion of the study population (n = 149, of which 76 where night-shift workers, 21 recently started with working night shifts (<2 years), and 55 were more experienced (≥5 years)) only one sample collected during a day session was available, and no dietary information was available. The majority of blood samples were collected in the winter, 41.5% of the study participants had a BMI ≥25, and 15.4% had a recent infection. Median age of the study population was 42 years (ranging from 19 to 65).

**Table 1:** Description of the study population (total number of individuals = 230, total number of observations = 355).

Variable			
N Recent night-workers (number of samples)	n = 36 (45 day-shift samples, 14 night-shift samples)		
N Experienced night workers (number of samples)	n = 104 (141 day-shift samples, 48 night-shift samples)		
N Day workers (number of samples)	n = 89 (107 day-shift samples)		
Season samples were collected^a^	287 winter; 62 summer		
	**Recent night workers**	**Experienced night workers**	**Day workers**
Mean age (sd)	30 (10)	42 (11)	44 (13)
Mean BMI (sd)	23.2 (3.9)	24.6 (4.1)	24.9 (4.2)
Percentage samples collected in winter^a^	85.7%	88.2%	91.0%
Percentage of participants with a recent infection^a^	8.8%	11.1%	14.0%
Mean years of night-shift work experience (sd)	1.3 (0.8)	18.0 (10.9)	-
*Chronotype*^a^			
Clearly morning person	15.8%	11.3%	24.1%
More morning than evening person	18.4%	30.2%	34.1%
More evening than morning person	31.6%	22.6%	19.8%
Clearly evening person	18.4%	16.0%	7.7%
No preference	15.8%	19.8%	14.3%

^a^ Missing data excluded from frequency table

### Analysis of sCD36

In the measurements collected during the day sessions ICC_worker_ was 29.2% among day workers and 37.0% among night workers, indicating that the majority of variation in sCD36 levels was not due to between-worker variation, but due to other factors such as day-to-day variation.

**Fig 1:**
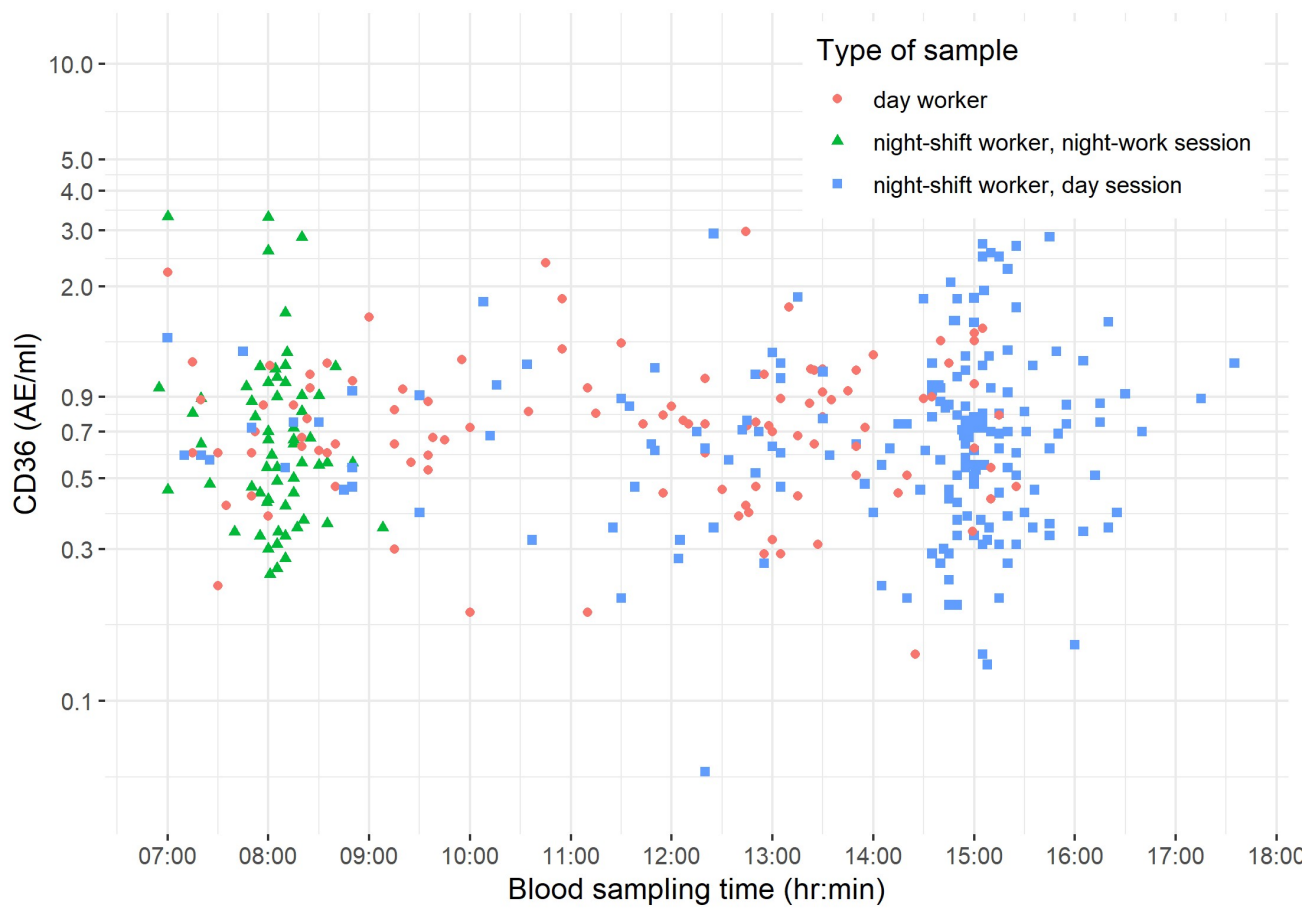
Log-transformed sCD36 levels in blood versus sampling times for night-shift workers during a night-shift session, night-shift workers during a day-shift session, and day workers during a day-shift session (n = 293 observations and 230 individuals).

We investigated if sCD36 levels were dependent of timing of blood sampling (wall-clock time). In Fig 1, we plot log-transformed sCD36 levels in blood versus sampling times for night-workers during a night shift session (samples collected between 06:55 and 09:08), night-workers during a day shift session (samples collected between 07:00 and 17:35), and day-workers during a day shift session (samples collected between 07:00 and 15:25). No clear patterns in timing of the sample collection and sCD36 levels were observed.

In addition, no clear pattern was found when we plotted sCD36 levels in blood versus the difference of timing in minutes between the last meal >50 kcal and timing of blood draw for night-workers during a night shift session and during a day shift session, and day-workers during a day shift session (S2 Appendix).

**Fig 2:**
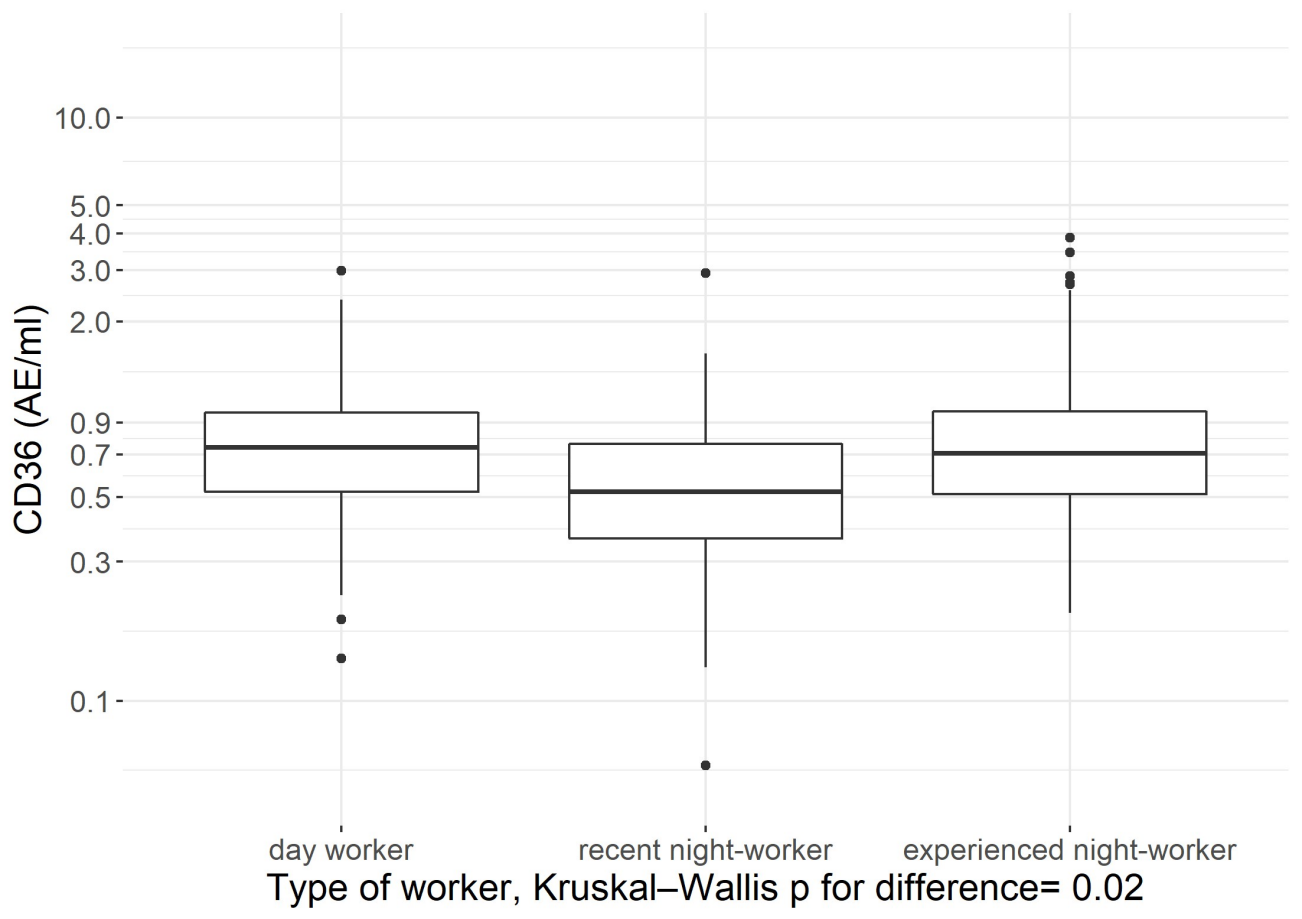
Log-transformed sCD36 levels in samples collected from day workers and night-shift workers during a day-shift session.

In Fig 2 plasma levels of sCD36 in samples collected from recent night-workers, experienced night-workers, and day workers during day shifts are presented. Levels assessed in samples from recent night-workers were lower than levels collected from experienced night workers and day workers (Kruskal-Wallis p-value of 0.02 for type of worker). In addition, we included a post-hoc Wilcoxon test assessing log-transformed sCD36 concentrations of recent night-shift workers versus day workers and experienced night-shift workers versus day workers, separately (S6 Appendix). Levels assessed in samples from recent night-workers were lower than levels collected from day workers (Wilcoxon p-value of 0.01 for type of worker). No differences were found between experienced night-shift workers and day workers (Wilcoxon p-value of 0.9 for type of worker).

In our main linear mixed model, we combined recent and experienced night-shift workers into one category and observed that night-shift workers had a slight non-significant increased level of sCD36 of 2.2% compared to day workers (CI of -12.6%, 19.4%, main model 1). This result did not change in any of the sensitivity analyses (Table 2). When only recent or experienced night-shift workers were compared to day workers, results were comparable to what we observed in Fig 1: a lower non-significant sCD36 level amongst recent night workers compared to day workers (-17.9%; 95% CI: -35.7% ─ 4.8%), while sCD36 levels among experienced night-shift workers were similar to day workers (5.2%; 95% CI: -10.5% ─ 23.8%). In addition, sCD36 levels were also similar between night-shift workers and day workers within a subset where control participants whom previously performed night work were excluded (5.0%, 95% CI: -13.8% - 28.0%, S4 Appendix. Analyses using a subset of controls whom did not previously worked night shifts). The estimates of the associations between the covariates and sCD36 from the multiple regression model, as shown in Table 2, are provided in S3 Appendix. Age was strongly associated with an increased sCD36 concentration (1.2%, 95% CI 0.5% - 1.9%).

**Table 2:** Percentage difference between day workers and night-shift workers in sCD36 concentrations in samples collected during a day-shift session.

	N obs	N ind	Night-shift worker versus day worker
Main model 1*	293	230	2.2% (-12.6%, 19.4%) p = 0.788
*Not corrected for BMI*	293	230	2.7% (-12.1%, 19.9%) p = 0.740
*Not corrected for chronotype*	293	230	2.2% (-12.2%, 18.9%) p = 0.779
*Additionally corrected for blood sampling time*	293	230	0.5% (-15.7%, 19.9%) p = 0.953
*Additionally corrected for meal timing*	147	84	-1.5% (-28.2%, 35.2%) p = 0.927
*Additionally corrected for time since waking up*	147	84	7.4% (-10.2%, 28.5%) p = 0.432
*Among morning types only*	135	103	-7.2% (-27.0%, 17.9%) p = 0.539
*Among evening types only*	95	76	-1.3% (-23.8%, 28.0%) p = 0.924
*Including only recent night workers*	152	126	-17.9% (-35.7%, 4.8%) p = 0.114
*Including only experienced night workers*	248	195	5.2% (-10.5%, 23.8%) p = 0.537
*Including only overweight individuals*	109	85	-1.8% (-23.9%, 26.8%) p = 0.889
*Excluding overweight individuals*	184	146	2.0% (-16.0%, 24.0%) p = 0.839

*corrected for age, BMI, recent infection, season, and chronotype.

N obs = number of observations

N ind = number of individuals

**Fig 3:**
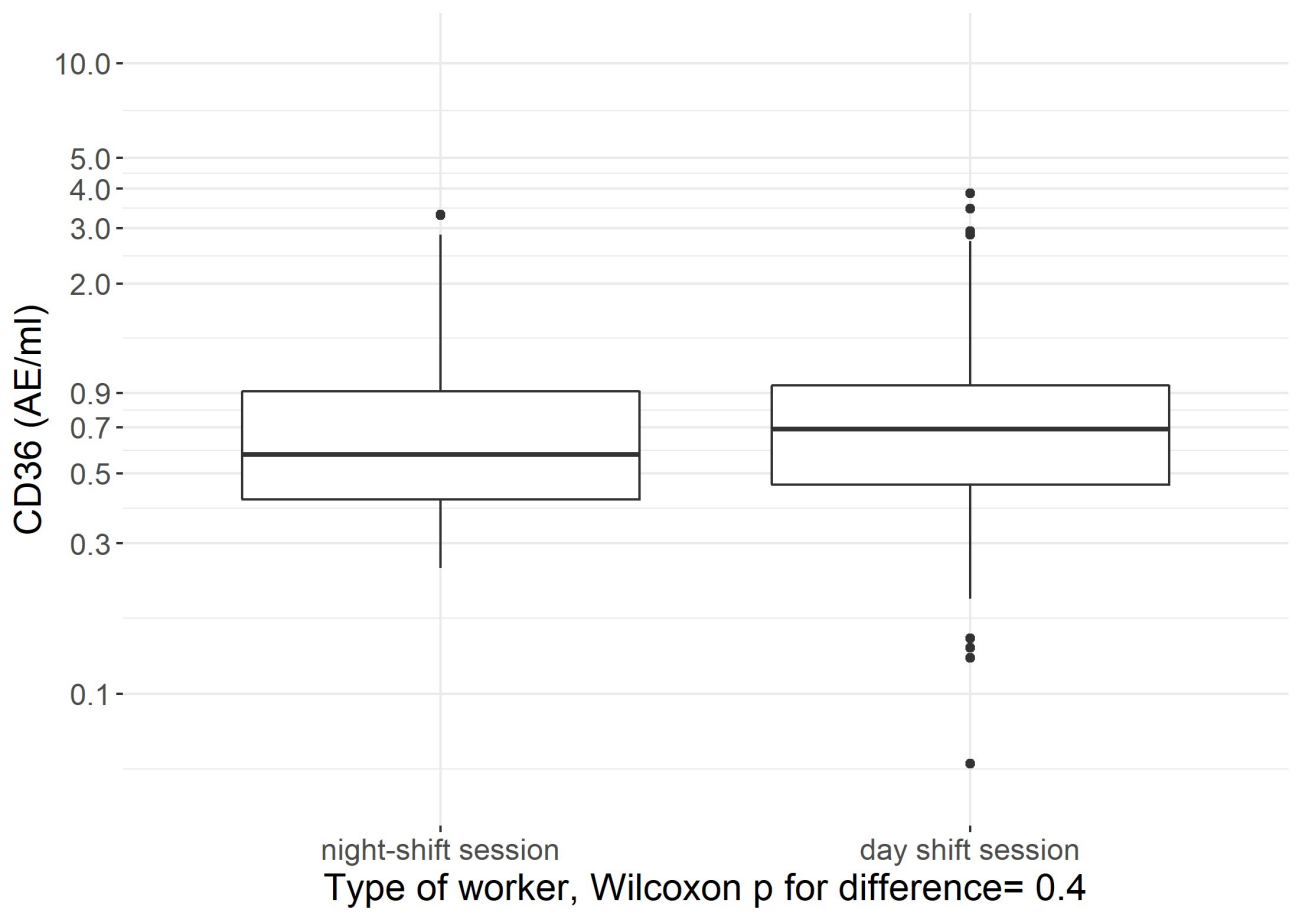
Log-transformed sCD36 levels in samples collected during night-shift sessions and day-shift sessions among night-shift workers.

In Fig 3 we describe sCD36 levels collected during a night-shift session and samples collected during a day shift session among night-shift workers to provide an indication if sCD36 is a marker for acute circadian disruption (Wilcoxon p-value 0.4; Fig 3). Results from linear-mixed models presented in Table 3 yield a similar inference, which did not substantially change in most of the sensitivity analyses. An exception was a borderline significant lower mean sCD36 level in night-shift samples collected among experienced night workers compared to day samples during a day shift of the same workers (-14.6%; 95% CI: -27.2%, -0.1%), while the mean sCD36 level among these samples in recent night workers was non-significantly elevated (29.9% (-14.0%, 96.3%) (Table 3). Additional analyses of night-shift work experience (duration and intensity) and CD36 concentration among night-shift workers are provided in S5 Appendix.

**Table 3:** Percentage difference in sCD36 concentrations between samples collected during a night-shift session and samples collected during a day-shift session among night-shift workers.

	N obs	N Ind	Night-shift session versus day shift session
Main model 2*	248	141	-7.8% (-20.7%, 7.1%) p = 0.287
*Not corrected for BMI*	248	141	-8.0% (-20.9%, 6.9%) p = 0.277
*Not corrected for chronotype*	248	141	-7.8% (-20.7%, 7.1%) p = 0.286
*Additionally corrected for blood sampling time*	248	141	-12.8% (-36.5%, 19.9%) p = 0.399
*Additionally corrected for meal timing*	174	67	-14.4% (-28.2%, 2.2%) p = 0.0862
*Additionally corrected for time since waking up*	174	67	-3.6% (-29.8%, 32.4%) p = 0.822
*Among morning types only*	99	53	-10.6% (-31.3%, 16.4%) p = 0.405
*Among evening types only*	90	52	4.1% (-18.3%, 32.8%) p = 0.744
*Among recent night workers only*	59	36	29.9% (-14.0%, 96.3%) p = 0.214
*Among experienced night workers only*	189	105	-14.6% (-27.2%, 0.1%) p = 0.0513
*Including only overweight individuals*	91	51	-14.1% (-33.2%, 10.4%) p = 0.235
*Excluding overweight individuals*	157	93	-6.7% (-22.8%, 12.7%) p = 0.471

*corrected for age, BMI, recent infection, season, and chronotype.

N obs = number of observations

N ind = number of individuals

## Discussion

In this study, we compared levels of plasma sCD36 of short-term (< 2 years), long-term (> 5 years) night-shift workers and day workers within the Klokwerk study. The results described in the current study do not provide indications for a relation between night-shift work experience (recent or long-term) and levels of sCD36 in plasma when the analysis was adjusted for potential confounding factors.

There was no immediate effect of a night shift on sCD36 levels measured in the blood the morning immediately after. There are some limitations of this study that could have influenced the results. The ICC_worker_ indicated that the variation in sCD36 levels between workers was only slightly higher than the variation from day-to-day, which makes it difficult to distinguish night-shift workers from day-workers in this analysis. Due to practical considerations, participants were not instructed to fast before blood sample collection. Previous data indicated an acute effect of food consumption on sCD36 levels [26]. We did not find such an effect in our data. The high level of variation from day-to-day was not explained by differences in moment of blood sampling with respect to meal timing. Among the day workers in the current study there was a high proportion of workers (56.1%) that have worked in night-shifts before they became day workers (more than 5 years ago). Considering our hypothesis of a long-term effect of shift-work on sCD36 levels, there is a possibility that such previous shift work experience influenced sCD36 levels in our study. The effects of external influences on sCD36 might however also be reversible, which was indicated in previous studies for some specific cases (following participants after weight loss and gastric bypass) [27,28]. Within sensitivity analyses among a subset without participants whom previously worked night shifts (S4 Appendix. Analyses using a subset of controls whom did not previously worked night shifts) we did not see stronger effects of night-shift work on sCD36 levels, but this might be because lack of power (only 39 control subject did never work night shifts).

Our rationale to split up short-term and long-term night-shift workers was to assess whether we could detect potential adaptation or a selection process, or cumulative effects among more experienced night-shift workers that might lead to a different response in the effect on CD36. As we did not see clear differences, we combined the experienced and less-experienced groups to increase statistical power in our analyses. The lack of differences between night-shift workers and day workers is in contrast with our previous study in mice, in which chronic circadian disturbance was related to an increase in sCD36 [10]. What contributes to this contrast in findings are the differences between the well-controlled and uniform mouse population and the heterogeneous human population—both in terms of heterogeneity among humans and the blood sampling in relation to exposure as well as the less standardized exposure. The exposure scenarios between the two studies were also different in the sense that in the current study within humans we compared sCD36 levels in real life, with less extreme exposure scenarios compared to the mice study. In addition, interspecies differences might play a role.

In our study sCD36 levels did not seem affected immediately after a night shift compared to a day shift either. sCD36 levels of experienced night workers seemed less affected by a night-shift session in terms of directions of effect compared to recent night workers, a possible indication for the bias known as ‘the healthy-worker effect’. Since this is a cross-sectional study, we cannot make sure shift workers stopped working night shifts due to health reasons, leaving a relatively healthy cohort of workers. The different direction of effect between experienced night workers and recent night workers might also indicate that night work experience might be a possible effect modifier in the association between night-shift work and sCD36. Perhaps the time span of previous night-shift work experience is relevant. In other words, night-shift work might be a cumulative exposure, which is visible after a certain number of years of night-shift work experience. As for the control group, time-since-exposure is also relevant, or the number of years since the last night shift before the effects on sCD36 levels are no longer visible. Since we do not know if and for which time span night-shift work is associated with changes in sCD36 levels, our study design may not be optimal and could play a role in our findings. Further evidence could be gathered in large cohort studies.

Our results do not provide evidence that sCD36 can be used as a biomarker for acute or chronic circadian disturbance in humans as we previously hypothesized. However, these results should be carefully interpreted and more research into this potential marker for chronic disturbance is required.

## Supporting information

S1 Appendix Analyses of missing data pattern. Figure A Percentage of missing cases for each variable reflected in a histogram and pattern of missing data. Almost 90% of the samples are not missing data. Figure B Missing data pattern for the variables BMI and chronotpe. The red box plot shows the distribution of missing data for chronotype while the blue box plot shows the distribution of the remaining data points.
(DOCX)

S2 Appendix Plotted sCD36 levels in blood versus the difference of timing in minutes between the last meal >50 kcal and timing of blood draw for night-workers and day-workers. Fig A Log-transformed sCD36 levels in blood versus difference in timing (min) between last meal and blood draw for night-shift workers during a night-shift session, night-shift workers during a day-shift session, and day workers during a day-shift session (n = 147 observations and 84 individuals).
(TIFF)

S3 Appendix Analyses of associations between covariates and CD36 concentration. Table A. Percentage difference in sCD36 concentrations in samples collected during a day-shift session for different covariates. *corrected for age, BMI, recent infection, season, and chronotype. **versus category: clearly morning person. N obs = number of observations N ind = number of individuals.
(DOCX)

S4 Appendix Analyses using a subset of controls whom did not previously worked night shifts. **Table A. Percentage difference between day workers and night-shift workers in sCD36 concentrations in samples collected during a day-shift session, subset without participants whom previously worked night shifts.** *corrected for age, BMI, recent infection, season, and chronotype. N obs = number of observations. N ind = number of individuals.
(DOCX)

S5 Appendix Analyses of night-shift work experience (duration and intensity) and CD36 concentration among night-shift workers. **Table A. Percentage difference between day workers and night-shift workers in sCD36 concentrations and shift-work duration (total years of night-shift work) in samples collected during a day-shift session among night-shift workers.** *corrected for age, BMI, recent infection, season, and chronotype. N obs = number of observations. N ind = number of individuals. **Table B. Percentage difference between day workers and night-shift workers in sCD36 concentrations and shift-work intensity (number of night shifts per month) in samples collected during a day-shift session among night-shift workers.** *corrected for age, BMI, recent infection, season, and chronotype. N obs = number of observations. N ind = number of individuals.
(DOCX)

S6 Appendix Post-hoc Wilcoxon test. Figure A Post-hoc Wilcoxon test assessing log-transformed sCD36 concentrations of recent night-shift workers versus day workers and experienced night-shift workers versus day workers.
(TIFF)
