# Supplementary figures and images for: Plasma sCD36 as non-circadian marker of chronic circadian disturbance in shift workers
Source: PLoS One. 2019 Oct 24;14(10):e0223522. doi: 10.1371/journal.pone.0223522 (PMC6812747; doi:10.1371/journal.pone.0223522)

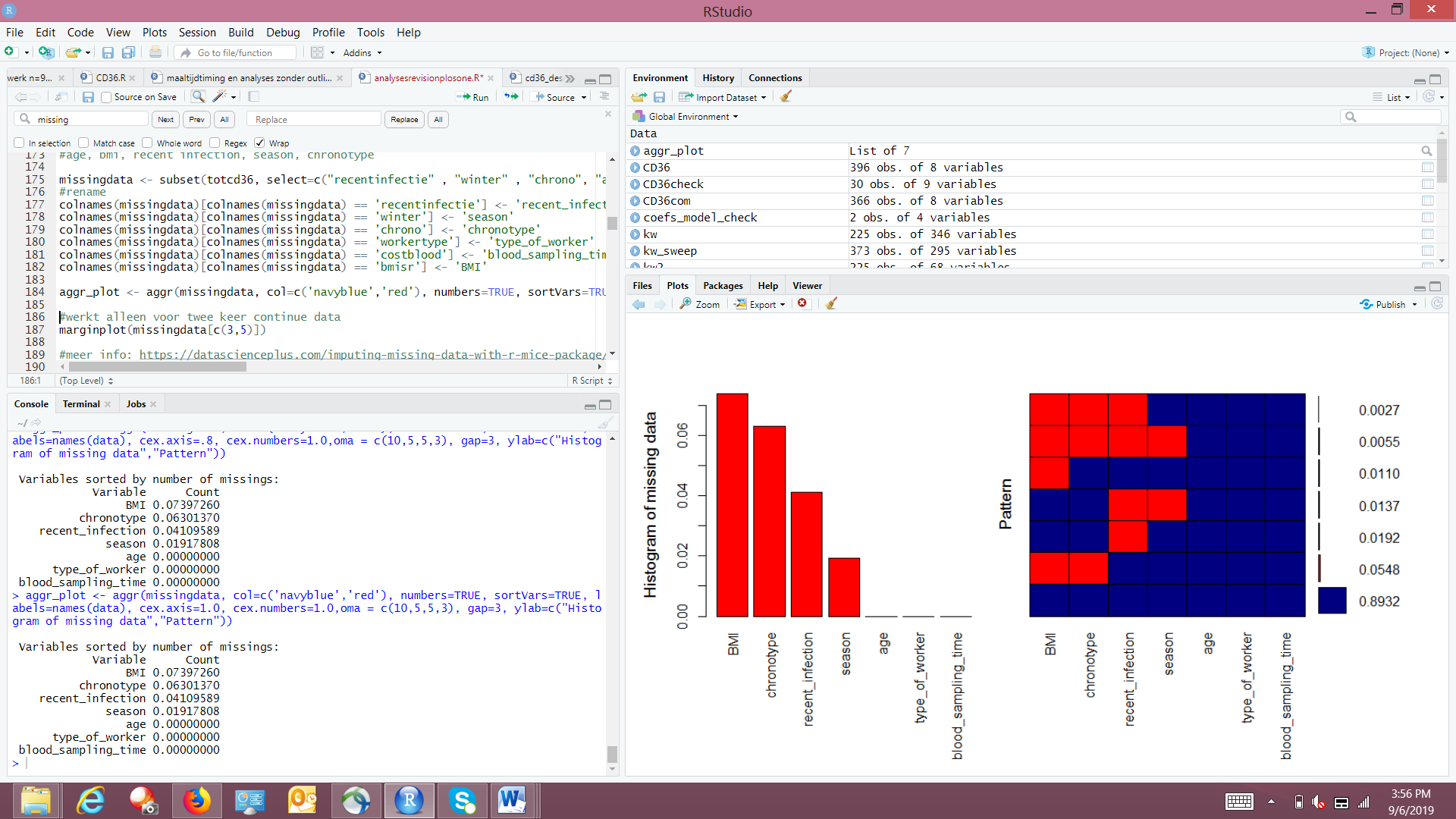


**Figure A**


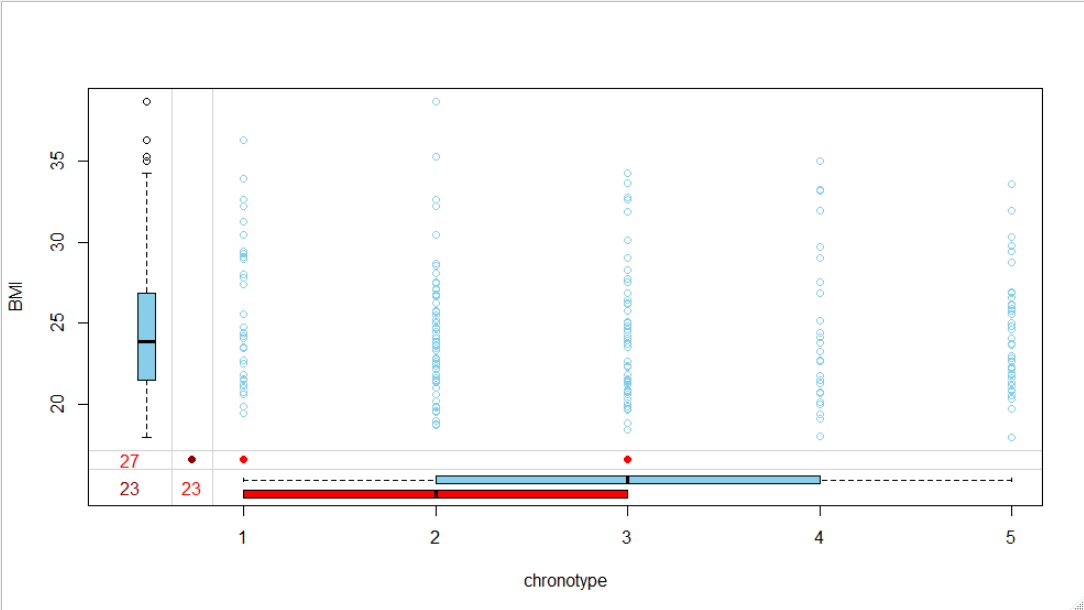


**Figure B**

Supplement: S1 Appendix — Figure A Percentage of missing cases for each variable reflected in a histogram and pattern of missing data. Almost 90% of the samples are not missing data. Figure B Missing data pattern for the variables BMI and chronotpe. The red box plot shows the distribution of missing data for chronotype while the blue box plot shows the distribution of the remaining data points. (DOCX) [file pone.0223522.s001.docx]

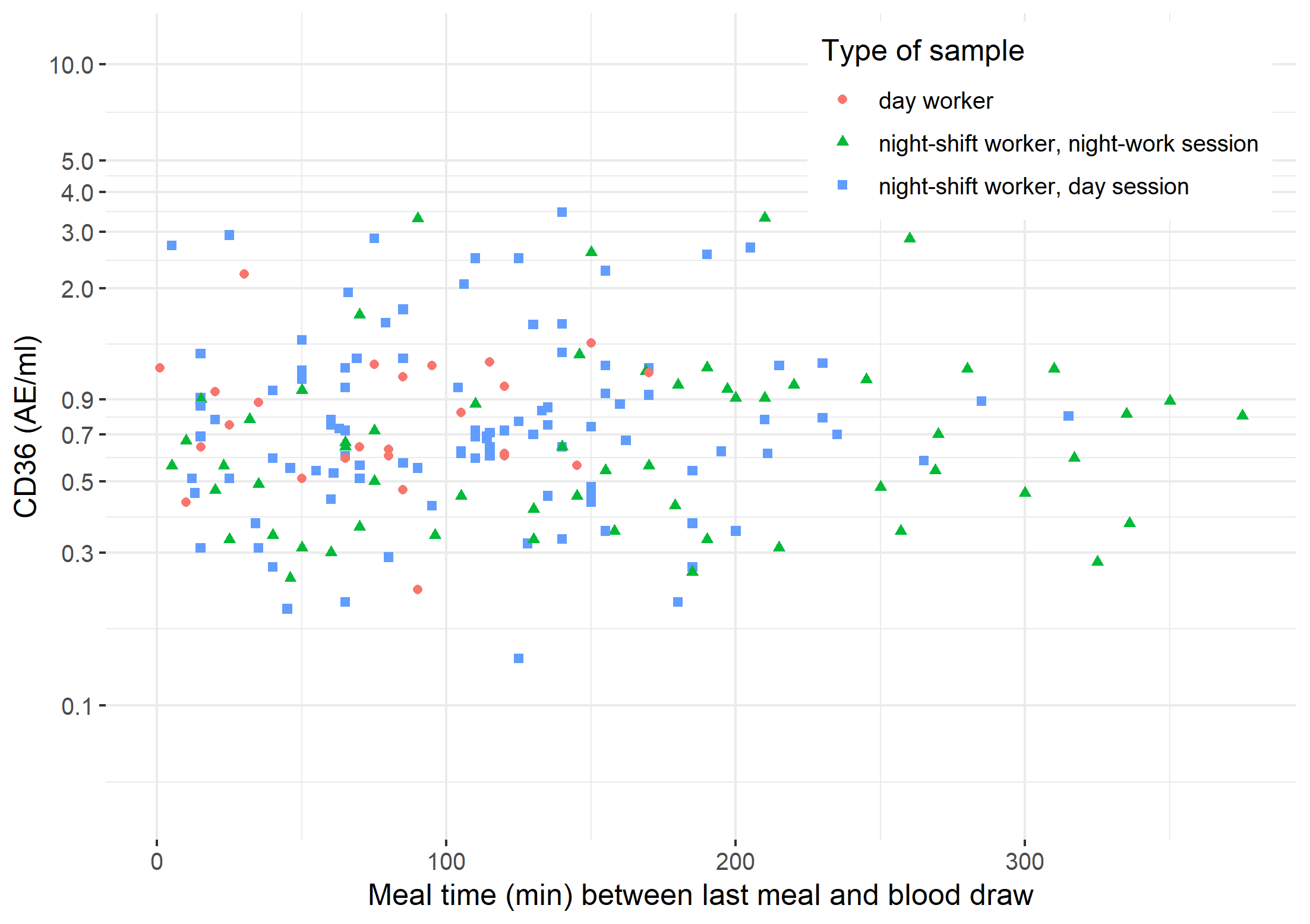

Supplement: S2 Appendix — Fig A Log-transformed sCD36 levels in blood versus difference in timing (min) between last meal and blood draw for night-shift workers during a night-shift session, night-shift workers during a day-shift session, and day workers during a day-shift session (n = 147 observations and 84 individuals). (TIFF) [file pone.0223522.s002.tiff]

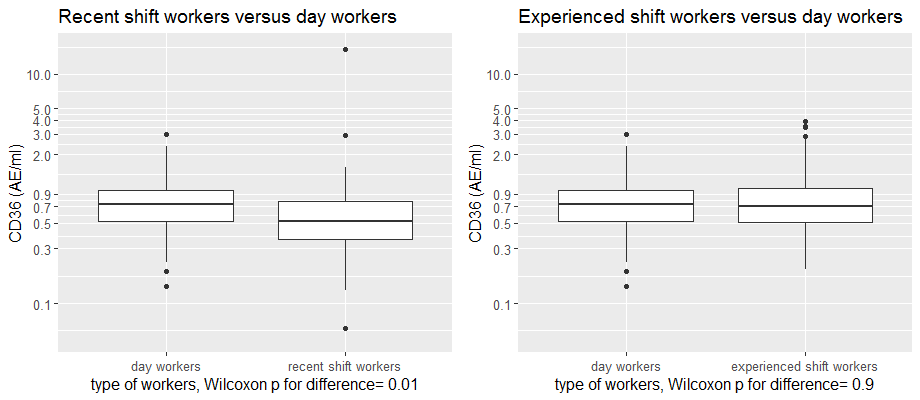

Supplement: S6 Appendix — Figure A Post-hoc Wilcoxon test assessing log-transformed sCD36 concentrations of recent night-shift workers versus day workers and experienced night-shift workers versus day workers. (TIFF) [file pone.0223522.s006.tiff]
