# Supplementary material for: Plasma sCD36 as non-circadian marker of chronic circadian disturbance in shift workers
Source: PLoS One. 2019 Oct 24;14(10):e0223522. doi: 10.1371/journal.pone.0223522 (PMC6812747; doi:10.1371/journal.pone.0223522)
Supplement: S3 Appendix — Table A. Percentage difference in sCD36 concentrations in samples collected during a day-shift session for different covariates. *corrected for age, BMI, recent infection, season, and chronotype. **versus category: clearly morning person. N obs = number of observations N ind = number of individuals. (DOCX) [file pone.0223522.s003.docx]

|  | N obs | N  ind | Estimate |
| --- | --- | --- | --- |
| *BMI* | 293 | 230 | -7.7% (-22.33% - 9.7%)  p = 0.364 |
| *Age* | 293 | 230 | 1.2% ( 0.5% - 1.9%)  p = 0.00125 |
| *Chronotype: more morning than evening person*** | 293 | 230 | 4.3% (-19.4% - 35.1%)  p = 0.747 |
| *Chronotype: more evening than morning person*** | 293 | 230 | -1.4% (-24.3% - 28.3%)  p = 0.914 |
| *Chronotype: clearly evening person*** | 293 | 230 | 1.5% (-27.7% - 34.2%)  p = 0.922 |
| *Chronotype: no preference*** | 293 | 230 | 8.8% (-17.7% - 43.9%)  p = 0.553 |
| *Blood sampling time* | 293 | 230 | 6.3% (-31.1% - 64.0%)  p = 0.782 |
| *Meal timing* | 147 | 84 | -0.03% ( -0.2% - 0.1%)  p = 0.602 |
| *Time since waking up* | 147 | 84 | 7.6% (-15.3% - 36.7%)  p = 0.549 |
| *Recent infection* | 293 | 230 | 1.6% (-19.6% - 28.5%)  p = 0.892 |
| *Season* | 293 | 230 | 2.5% (-18.6% - 29.2%)  p = 0.832 |

*corrected for age, BMI, recent infection, season, chronotype, and shift-worker type (night-shift worker versus day worker).

**versus category: clearly morning person.

N obs = number of observations

N ind = number of individuals
