# Supplementary material for: Plasma sCD36 as non-circadian marker of chronic circadian disturbance in shift workers
Source: PLoS One. 2019 Oct 24;14(10):e0223522. doi: 10.1371/journal.pone.0223522 (PMC6812747; doi:10.1371/journal.pone.0223522)
Supplement: S4 Appendix — Table A. Percentage difference between day workers and night-shift workers in sCD36 concentrations in samples collected during a day-shift session, subset without participants whom previously worked night shifts. *corrected for age, BMI, recent infection, season, and chronotype. N obs = number of observations. N ind = number of individuals. (DOCX) [file pone.0223522.s004.docx]

|  | N obs | N  ind | Night-shift worker versus day worker |
| --- | --- | --- | --- |
| Main model 1* | 242 | 191 | 5.0% (-13.8%, 28.0%)  p = 0.627 |
| *Not corrected for BMI* | 242 | 191 | 5.9% (-13.1%, 28.9%)  p = 0.570 |
| *Not corrected for chronotype* | 242 | 191 | 5.2% (-13.3%, 27.6%)  p = 0.608 |
| *Additionally corrected for blood sampling time* | 242 | 191 | 3.6% (-16.5%, 28.6%)  p = 0.747 |
| *Additionally corrected for time since waking up* | 242 | 191 | 10.5% (-11.1%, 37.4%)  p = 0.367 |
| *Among morning types only* | 100 | 79 | -5.0% (-31.0%, 30.8%)  p = 0.754 |
| *Among evening types only* | 87 | 68 | 0.1% (-25.0%, 33.6%)  p = 0.996 |
| *Including only recent night workers* | 100 | 87 | -16.4% (-37.8%, 12.5%)  p = 0.238 |
| *Including only experienced night workers* | 197 | 156 | 8.7% (-11.3%, 33.2%)  p = 0.422 |
| *Including only overweight individuals* | 84 | 65 | 6.1% (-22.5%, 45.2%)  p = 0.713 |
| *Excluding overweight individuals* | 158 | 126 | 0.6% (-21.9%, 29.0%)  p = 0.963 |

*corrected for age, BMI, recent infection, season, and chronotype.

N obs = number of observations

N ind = number of individuals
