# Supplementary material for: Plasma sCD36 as non-circadian marker of chronic circadian disturbance in shift workers
Source: PLoS One. 2019 Oct 24;14(10):e0223522. doi: 10.1371/journal.pone.0223522 (PMC6812747; doi:10.1371/journal.pone.0223522)
Supplement: S5 Appendix — Table A. Percentage difference between day workers and night-shift workers in sCD36 concentrations and shift-work duration (total years of night-shift work) in samples collected during a day-shift session among night-shift workers. *corrected for age, BMI, recent infection, season, and chronotype. N obs = number of observations. N ind = number of individuals. Table B. Percentage difference between day workers and night-shift workers in sCD36 concentrations and shift-work intensity (number of night shifts per month) in samples collected during a day-shift session among night-shift workers. *corrected for age, BMI, recent infection, season, and chronotype. N obs = number of observations. N ind = number of individuals. (DOCX) [file pone.0223522.s005.docx]

|  | N obs | N  ind | Night-shift worker versus day worker |
| --- | --- | --- | --- |
| Main model 1* | 248 | 141 | 0.65% ( -0.7% - 2.0%)  p = 0.355 |
| *Not corrected for BMI* | 248 | 141 | 0.62% ( -0.7% - 2.0%)  p = 0.373 |
| *Not corrected for chronotype* | 248 | 141 | 0.77% ( -0.6% - 2.1%)  p = 0.261 |
| *Additionally corrected for blood sampling time* | 248 | 141 | 0.77% ( -0.5% - 2.1%)  p = 0.251 |
| *Additionally corrected for time since waking up* | 174 | 67 | -0.06% ( -1.7% - 1.6%)  p = 0.944 |
| *Among morning types only* | 99 | 53 | 0.78% ( -1.1% - 2.7%)  p = 0.418 |
| *Among evening types only* | 90 | 52 | 0.16% ( -2.5% - 2.9%)  p = 0.910 |
| *Including only recent night workers* | 59 | 36 | -19.09% (-42.2% - 13.2%)  p = 0.216 |
| *Including only experienced night workers* | 189 | 105 | 0.34% ( -1.4% - 2.1%)  p = 0.700 |
| *Including only overweight individuals* | 91 | 51 | 0.54% ( -1.5% - 2.6%)  p = 0.608 |
| *Excluding overweight individuals* | 157 | 93 | 0.59% ( -1.2% - 2.4%)  p = 0.525 |

Table A

*corrected for age, BMI, recent infection, season, and chronotype.

N obs = number of observations

N ind = number of individuals

**Table B**

|  | N obs | N  ind | Night-shift worker versus day worker |
| --- | --- | --- | --- |
| Main model 1* | 248 | 141 | 1.45% ( -2.2% - 5.2%)  p = 0.441 |
| *Not corrected for BMI* | 248 | 141 | 0.94% ( -2.5% - 4.5%)  p = 0.595 |
| *Not corrected for chronotype* | 248 | 141 | 2.1% ( -1.5% - 5.7%)  p = 0.256 |
| *Additionally corrected for blood sampling time* | 248 | 141 | 0.16% ( -2.9% - 3.3%)  p = 0.917 |
| *Additionally corrected for time since waking up* | 174 | 67 | 0.76% ( -2.9% - 4.5%)  p = 0.686 |
| *Among morning types only* | 99 | 53 | -1.2% ( -7.0% - 5.0%)  p = 0.703 |
| *Among evening types only* | 90 | 52 | -1.05% ( -5.2% - 3.3%)  p = 0.627 |
| *Including only recent night workers* | 59 | 36 | -0.18% ( -6.9% - 7.0%)  p = 0.960 |
| *Including only experienced night workers* | 189 | 105 | 0.21% ( -3.2% - 3.7%)  p = 0.905 |
| *Including only overweight individuals* | 91 | 51 | -0.56% ( -5.2% - 4.3%)  p = 0.818 |
| *Excluding overweight individuals* | 157 | 93 | -0.83% ( -4.727% - 3.2%)  p = 0.682 |

*corrected for age, BMI, recent infection, season, and chronotype.

N obs = number of observations

N ind = number of individuals
